# Impact of cooling garments on performance during and after vigorous, heart‐rate‐clamped exercise in young males under hot and humid conditions

**DOI:** 10.14814/phy2.70898

**Published:** 2026-04-29

**Authors:** Kazutaka Ota, Hiroaki Tamaru, Kazushige Sasaki

**Affiliations:** ^1^ Department of Life Sciences, Graduate School of Arts and Sciences The University of Tokyo Tokyo Japan; ^2^ Japan Sport Council Tokyo Japan

**Keywords:** cycle ergometer, fan‐attached garment, sugar alcohol, temperature, thermal sensation

## Abstract

This study aimed to clarify the effects of the following cooling garments on performance during and after vigorous, heart‐rate‐clamped exercise under hot and humid conditions: base layers made of cross‐shaped fibers (C), sugar alcohol‐printed base layers (S), and a combination of S with a fan‐attached jacket (S+F). Fifteen healthy male participants wore the cooling garments and rested for 20 min in a room set to ~30°C and ~60% relative humidity. The participants then completed a 20‐min cycle ergometer exercise with heart rate clamped at 65% of heart rate reserve and rated their perceived exertion (RPE). Before and after exercise, we assessed thermal, comfort, and wetness sensations and measured body temperature, vertical jump height, ground reaction force during rising from the chair, visual reaction time, and the Stroop interference. Cooler sensations were consistently reported in the order of S+F, S, and C. Despite the lowest RPE, pedaling load was highest in S+F. Sweat loss was comparable among the conditions, while garment sweat absorption and post‐exercise skin temperature were lowest in S+F. These results suggest that S+F improves endurance performance under hot and humid conditions through efficient evaporative heat loss mainly facilitated by increased airflow from the fans.

## 1 INTRODUCTION

Global warming has increased the risk of environmental heat stress through more frequent and severe climate extremes, posing serious health risks such as dehydration, heat exhaustion, and heat stroke. In the context of exercise or physical work, endurance performance is negatively affected by hot environments (Cheuvront et al., 2010; Périard et al., 2021). To dissipate excess heat through conduction, convection, radiation, and evaporation, autonomic responses such as cutaneous vasodilation (Trangmar & González‐Alonso, 2025) and sweating (Nadel et al., 1971) are triggered. These responses can lead to a decrease in stroke volume, placing additional strain on cardiovascular function (Périard et al., 2021; Rowell, 1974).

To support physical activity in the heat, various body cooling strategies have been developed. Pre‐cooling methods such as cold‐water immersion, cold‐water ingestion, and ice pack application are known to improve endurance performance (Bongers et al., 2021; Tyler et al., 2015). However, their cooling benefits typically fade within ~25 min after the onset of exercise (Bolster et al., 1999), which underscores the importance of per‐cooling strategies applied during exercise. Several per‐cooling approaches such as cooling garments, neck coolers, cold drinks, and ice slurries have been shown to improve endurance performance in the heat, with cooling garments being particularly effective (Bongers et al., 2021; Périard et al., 2021; Stevens et al., 2017).

Various types of cooling garments have been developed and studied (Havenith, 2002; Périard et al., 2021). Garments incorporating phase change materials (e.g., ice and paraffin) and cold‐water perfusion systems have been shown to suppress excessive increases in body temperature and heart rate, thereby enhancing endurance performance (Bach et al., 2019). However, these garments have several disadvantages, including the need for pre‐cooling, bulkiness, and limited cooling duration, which make them impractical for continuous use in athletic settings (Xu et al., 2024). Moreover, they restrict sweat evaporation, potentially causing increased discomfort in humid environments (Xu et al., 2024).

To overcome these challenges, a novel type of cooling garment has been developed that utilizes the endothermic dissolution of sugar alcohols (e.g., xylitol and erythritol) printed on the fabric. Although their skin cooling effects were demonstrated using nanofiber mats (Yang et al., 2024), garment‐based studies have so far been limited to a combination with phase change materials and to a comparison exclusively with non‐cooling garments (McFarlin et al., 2016). Therefore, it remains unexplored whether sugar alcohol‐printed garments outperform other cooling garments during vigorous physical activity in the heat.

To address this gap, the present study involves comparing sugar alcohol‐printed garments with a different type of cooling garment made of cross‐shaped fibers (Yang et al., 2021). In addition, this study addresses the combined effect of the sugar alcohol‐printed garments with a fan‐attached garment, as the drying of the fabric enhanced by airflow may allow for repeated endothermic dissolution of sugar alcohols. Although fan‐attached garments have been shown to reduce thermal strain during very light‐to‐moderate physical activity (Hadid et al., 2008; Hashimoto et al., 2021; Mori et al., 2022; Xu et al., 2025), their efficacy during vigorous activities, common in many sports, remains unclear. Moreover, previous studies have largely focused on subjective fatigue, with little attention to post‐exercise changes in physical or cognitive performance. This is an important limitation, as heat stress and dehydration induce not only subjective fatigue but also impairments in physical and cognitive performance (Périard et al., 2021). Cognitive impairments are particularly relevant in occupational and athletic settings, given that they can compromise both performance and safety even during post‐exercise, non‐physical tasks.

Therefore, this study aimed to investigate the effects of different cooling garment conditions on physical and cognitive performance under hot and humid conditions. For this purpose, participants performed vigorous‐intensity, heart‐rate‐clamped cycle ergometer exercises on separate days while wearing one of the following: base layers made of cross‐shaped fibers (C), sugar alcohol‐printed base layers (S), and S combined with a fan‐attached jacket (S+F). Physical and cognitive performance was assessed before and after exercise via vertical jump, chair stand, visual reaction, and the Stroop tests, along with subjective ratings for thermal comfort. We hypothesized that S would be superior to C in terms of thermal comfort and performance maintenance, and that its benefits would be synergistically enhanced with a fan‐attached jacket.

## 2 METHODS

### 2.1 Ethical approval

This study was performed in accordance with the principles of the *Declaration of Helsinki* without being registered. Approval was granted by the Ethical Review Committee for Experimental Research involving Human Subjects, Graduate School of Arts and Sciences, The University of Tokyo (approval number: 1020). All the participants were informed of the procedures and possible risks and gave their written informed consent prior to the experiment.

### 2.2 Participants

Fifteen healthy male volunteers, ranging from recreationally active individuals to competitive athletes, participated in this study. Their mean (SD) age, height, body mass, and body mass index were 23.1 (1.8) years, 176.7 (5.2) cm, 71.3 (11.4) kg, and 22.8 (3.2) kg m^−2^, respectively. The volunteers were free from any neuromuscular disorder, pathology, or surgery. A priori sample size calculation was performed with G*Power version 3.1.9.6. (Heinrich Heine Universität Düsseldorf, Germany) using a within‐participants analysis of variance (ANOVA) with a statistical power of 0.8 and an alpha error of 0.05. Twelve participants were required to detect an effect size (Cohen's *f*) of 0.4, which is considered large (Cohen, 1988). We increased the number to fifteen, considering potential participant dropout due to heat stress.

### 2.3 Experimental design

**FIGURE 1:**
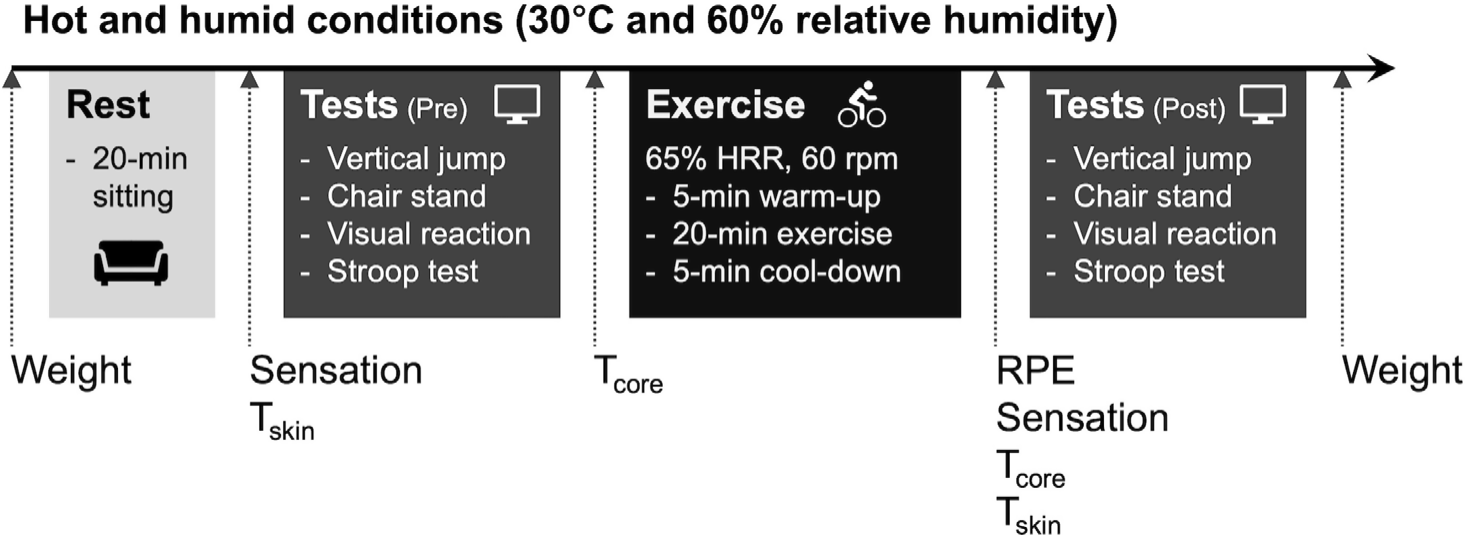
Experimental procedure. All procedures were conducted in a room set to ~30°C and ~60% relative humidity. After 20 min of seated rest, a series of physical and cognitive tests was performed. Following a cycle ergometer exercise with heart rate clamped at 65% of heart rate reserve (HRR), the same tests were repeated. Before and after exercise, the following data were collected: weights (body, garments, and bottled water), skin temperature (T_skin_), core temperature (T_core_), and subjective sensations (thermal, comfort, and wetness). The rate of perceived exertion (RPE) was measured only immediately after the exercise.

The present study used a within‐participants design. Each participant visited our laboratory four times, with at least 24 h between visits. To minimize the potential influence of diurnal variations in body temperature and motor performance, all experiments were conducted during the daytime (between 10:30 and 17:00). The participants were instructed to keep the start time consistent across days whenever possible. Nevertheless, an inter‐day variation in start time occurred within participants (33 [34] min), as several participants did not fully comply with this instruction. In the first visit, participants familiarized themselves with cycle ergometer exercise up to vigorous intensity and a series of physical and cognitive performance tests. The number of trials for each test was identical to that used in subsequent experiments. In the second to fourth visits, the participants completed the physical and cognitive performance tests before and after the cycle ergometer exercise (Figure 1) under the three different garment conditions in a hot and humid environment. The order of garment conditions was randomized across participants. Participants were asked to avoid any strenuous exercise 24 h before visiting the laboratory.

Room temperature and humidity were continuously monitored using a thermohygrometer (608‐H1, Testo, Germany) and controlled at target values of 30°C and 60% relative humidity using air conditioners and humidifiers. The actual environmental conditions are reported in the Results section. Upon the participants' arrival, body mass was measured using a force plate (TF6090, Tec Gihan, Japan). The weights of all assigned garments and a commercially available bottled water were then measured using a digital weighing scale (CS‐20KWP, Custom, Japan). Participants then put on the assigned garments and were provided with the bottled water for ad libitum consumption. After 20 min of seated rest, subjective sensations and skin temperatures were measured. After a series of physical and cognitive performance tests, core temperature was measured on the forehead (see below). Immediately after 20‐min cycle ergometer exercise, the rate of perceived exertion (RPE) was measured with a 6–20 Borg category scale (Borg, 1982). Subsequently, the measurements of body temperatures and subjective sensations, along with the performance tests, were repeated. Finally, body, all garments, and bottled water were weighed again to calculate water intake, sweat loss, and garment sweat absorption.

### 2.4 Cooling garments

**FIGURE 2:**
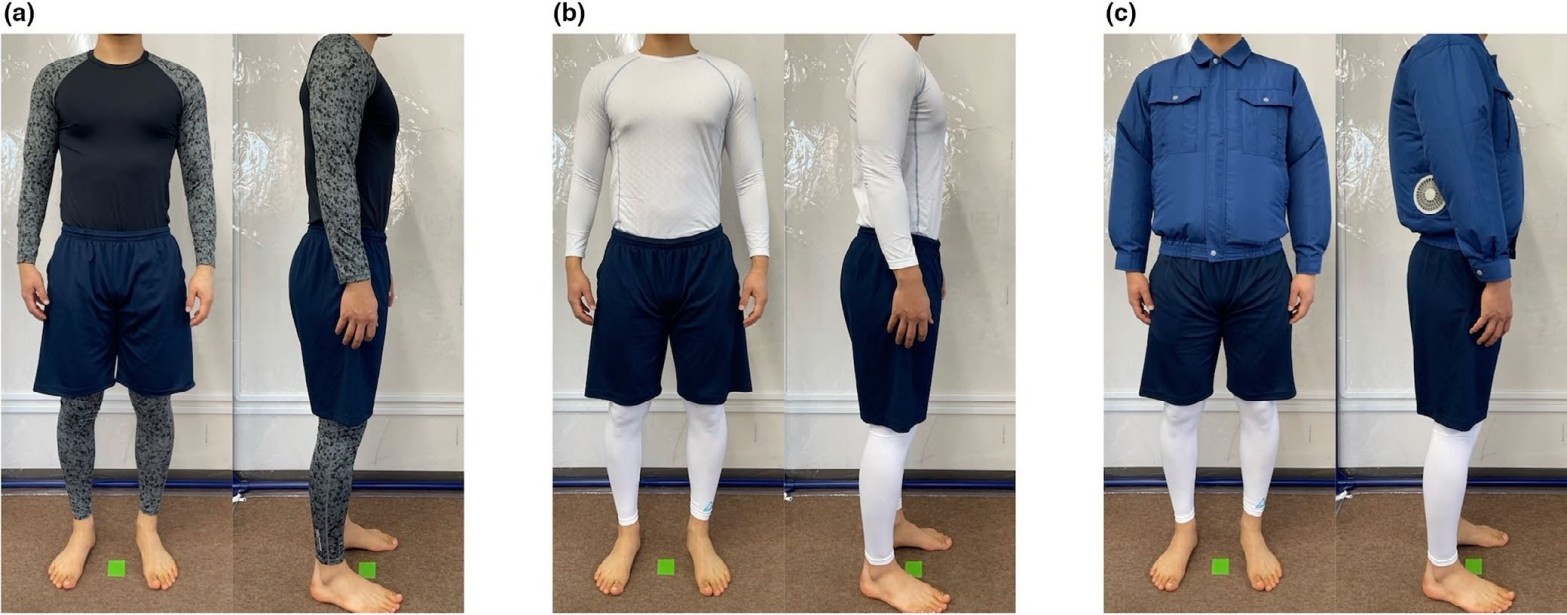
Cooling garments. (a) C condition: Participants wore base layers (a long‐sleeve shirt and full‐length tights) made of cross‐shaped fibers. (b) S condition: Participants wore sugar alcohol‐printed base layers. (c) S+F condition: Participants wore sugar alcohol‐printed base layers and a jacket equipped with two fans. In all conditions, participants also wore disposable briefs and athletic shorts underneath and over the base layers, respectively.

The participants wore base layers made of cross‐shaped fibers (a long‐sleeve shirt and full‐length tights, 85% polyester and 15% polyurethane, JW‐623 and JW‐632, Otafuku Glove, Japan) in C condition and sugar alcohol‐printed base layers (a long‐sleeve shirt and full‐length tights, 79% nylon and 21% polyurethane, Freeze Tech, Liberta, Japan) in both S and S+F conditions (Figure 2). They also wore disposable briefs (Hasocare, Haso, Japan) and athletic shorts (100% polyester, 00325‐ACP, Toms, Japan) underneath and over these base layers, respectively. In S+F condition, they additionally wore a jacket (100% polyester, KU90510, Kuchofuku, Japan) equipped with two fans (10‐cm diameter, 103 g, FAN2200, Kuchofuku) located near the left and right sides of the lower back, powered by a rechargeable Li‐ion battery (254 g, BTUL1, Kuchofuku). The fans were set to deliver airflow through the cuffs, collar, and hem at the maximum flow rate (48.8 L s^−1^).

### 2.5 Cycle ergometer exercise

The participants performed exercise with a cycle ergometer (75XLIII, Konami Sports, Japan). Just before the beginning of exercise, resting heart rate was measured using an ear sensor integrated into the ergometer. A target heart rate corresponding to 65% of heart rate reserve, classified as vigorous intensity (Garber et al., 2011), was then calculated as follows (Tanaka et al., 2001):

Maximum heart rate=208−0.7×age

$$\begin{array}{c} \text{Target heart rate}=0.65\times \left(\text{maximum heart rate}\right.\\ \left.\;-\text{resting heart rate}\right)\\ \;+\text{resting heart rate} \end{array}$$

The pedaling load was gradually increased until the heart rate reached the target value during a 5‐min warm‐up, maintained for 20 min, and then reduced to 20 W during a 5‐min cool‐down. The participants were instructed to maintain a pedaling cadence of 60 rpm. The pedaling load and heart rate were recorded every minute during the 20‐min main exercise, excluding the warm‐up and cool‐down periods, where the mean values were calculated for analysis.

### 2.6 Body temperature

Skin temperature was measured at seven sites (forehead, left arm, left hand, left foot, left leg, left thigh, and trunk) using an infrared thermometer (Thermofocus Pro, Technimed, Italy). Mean skin temperature was calculated according to the following formula (Hardy et al., 1938):

$$\begin{array}{c} \text{Mean skin temperature}=T_{\text{forehead}}\times 0.07+T_{\mathrm{arm}}\times 0.14\\ +T_{\text{hand}}\times 0.05+T_{\text{foot}}\times 0.07+T_{\mathrm{leg}}\times 0.13\\ +T_{\text{thigh}}\times 0.19+T_{\text{trunk}}\times 0.35 \end{array}$$

where T[site] represents the skin temperature at each site. Core temperature was measured with a zero‐heat‐flow method (Asai et al., 2023). Specifically, a temperature‐compensated probe (PD‐1, Terumo, Japan) connected to an electronic thermometer (CM‐210, Terumo) was placed on the forehead, with which the temperature 10 mm below the skin surface can be measured.

### 2.7 Subjective sensations

Thermal, comfort, and wetness sensations were measured with categorical rating scales ranging from −2 to 9 (−2, slightly cool; 0, neutral; 2, slightly warm; 4, warm; 6, hot; 8, very hot), 1 to 8 (1, neutral; 3, a little uncomfortable; 5, uncomfortable; 7, very uncomfortable), and −2 to 7 (−2, slightly dry; 0, neutral; 2, slightly wet; 4, wet; 6, very wet), respectively (Chou et al., 2011).

### 2.8 Vertical jump test

The participants were instructed to perform countermovement vertical jumps with maximal effort on a mat switch (T.K.K.1264p, Sanka, Japan). Starting from an upright position with their hands on their hips to prevent arm swing, they performed three trials with ~30‐s rest intervals. Jump height was calculated from flight time, measured from take‐off to landing (Bosco et al., 1983). The highest value among the three trials was used for analysis.

### 2.9 Chair stand test

The participants sat on a 40‐cm‐high chair with their arms crossed in front of the chest, trunk upright, ankles at 90°, and feet placed shoulder‐width apart on the force plate (TF6090), with which ground reaction force was recorded at 1000 Hz. They were instructed to stand up as fast as possible without countermovement. Three trials were performed with ~30‐s rest intervals. In addition to the peak force, the rate of force development (RFD) was calculated as the peak value of the first derivative of the ground reaction force, computed across overlapping 90‐ms windows (Tsuji et al., 2015). The highest peak force among the three trials and the RFD from the same trial were normalized to the corresponding body mass measured before and after cycle ergometer exercise and used for analysis.

### 2.10 Visual reaction test

The participants were instructed to adopt a standardized starting position on the mat switch (T.K.K.1264p), with their hands on their hips, feet shoulder‐width apart, and hips and knees slightly flexed. They were then required to lift both feet off the mat as quickly as possible in response to a visual stimulus from a light device connected to the mat switch. Three trials were performed with ~30‐s rest intervals. Foot contact signal was obtained from the mat switch. Surface electromyography (EMG) signals were recorded from the right vastus lateralis and medial gastrocnemius muscles using a wireless EMG system (Pico EMG, Cometa, Italy). All signals were digitized at 1000 Hz using a data acquisition system (PowerLab/16SP, ADInstruments, New Zealand). Offline analyses were performed using LabChart version 8.1.30 (ADInstruments) and Excel version 16.98 (Microsoft, USA). Reaction time was defined as the interval from stimulus onset to take‐off. Premotor time was defined as the interval from stimulus onset to EMG onset, identified from each muscle as the point where the signal exceeded three SD above baseline. The shortest reaction time and the corresponding premotor times were used for analysis.

### 2.11 Stroop test

The Stroop test (Stroop, 1935) was administered using PsyToolkit experimental library (Stoet, 2017). The participants were instructed to identify the ink color of words that were either congruent or incongruent with their semantic meaning (e.g., the word “blue” printed in red ink). The protocol included two blocks separated by a ~30‐s rest interval, each consisting of 40 visual stimuli presented in random order. To evaluate both speed and accuracy, the inverse efficiency score (IES) was calculated by dividing the mean response time by the proportion of correct responses across both congruent and incongruent trials within each block (Lee et al., 2025). The Stroop interference IES, an index of executive function performance, was calculated as the difference in IES between incongruent and congruent trials (Lee et al., 2025). Of the two blocks, the lower (better) IES and the corresponding interference IES were used for analysis.

### 2.12 Statistics

Data are expressed as means and SD. Statistical analyses were carried out using R version 4.3.2 (R Foundation for Statistical Computing, Austria) and the R function “anovakun” version 4.8.9 (Iseki, 2025). Differences in means across the three garment conditions (e.g., pedaling load, RPE, and sweat loss) were analyzed with a one‐way repeated‐measures ANOVA. For the body temperatures, subjective sensations, and performance data, a two‐way repeated‐measures ANOVA was used to test whether the effect of time (before vs. after exercise) differed among garment conditions. When a significant main effect or time‐by‐condition interaction was found, post hoc multiple comparisons were conducted using paired *t*‐tests with *p* values adjusted by the false discovery rate method (Benjamini & Hochberg, 1995). Generalized *η*‐squared (*η*_*G*_^2^) and Cohen's *d* were reported as measures of effect size for within‐participants ANOVA and paired *t*‐tests, respectively. A *p* value of <0.05 was considered statistically significant. Intra‐class correlation coefficient (ICC: type 3, 1) and standard error of measurement (SEM) were calculated as indicators of between‐day reliability (resting heart rate, body mass, body temperature, and physical and cognitive performance) and within‐day repeatability (physical and cognitive performance) of pre‐exercise measurements.

## 3 RESULTS

### 3.1 Ambient conditions

The overall mean (SD) room temperature and relative humidity at the beginning of each experiment were 30.6 (0.5)°C and 60.7 (1.4) %, respectively. The ambient conditions were well controlled, with no significant difference among the three garment conditions (one‐way ANOVA, temperature, *η*_*G*_^2^ = 0.013, *p* = 0.753; humidity, *η*_*G*_^2^ = 0.051, *p* = 0.379).

### 3.2 Cycle ergometer performance

**FIGURE 3:**
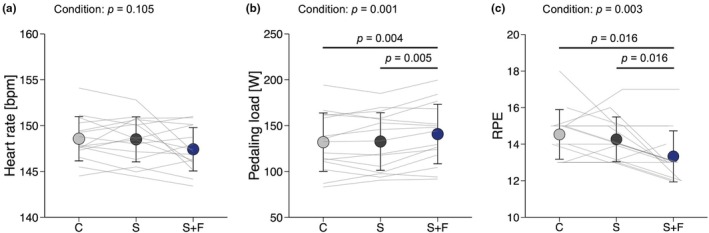
Cycle ergometer performance. (a) Mean heart rate and (b) pedaling load during 20‐min cycle ergometer exercise. (c) Rate of perceived exertion (RPE) immediately after cycle ergometer exercise. Data are presented as means and SD with individual line plots (*n* = 15). C, S, and S+F represent wearing base layers made of cross‐shaped fibers, sugar alcohol‐printed base layers, and a combination of S with a fan‐attached jacket, respectively. Data were analyzed with one‐way repeated‐measures analysis of variance followed by paired *t*‐tests with *p* values adjusted by the false discovery rate method.

The mean (SD) resting heart rate just before exercise was 66.9 (8.5) bpm in C, 65.7 (7.9) bpm in S, and 65.0 (7.1) bpm in S+F, which was not significantly different among the three garment conditions (*η*_*G*_^2^ = 0.010, *p* = 0.399). The ICC and SEM for the resting heart rate measured over 3 days were 0.75 and 3.9 bpm, respectively. Figure 3 shows the results for cycle ergometer performance. Significant main effects of condition were found for pedaling load (*η*_*G*_^2^ = 0.017, *p* = 0.001) and RPE (*η*_*G*_^2^ = 0.139, *p* = 0.003), but not for heart rate (*η*_*G*_^2^ = 0.049, *p* = 0.105). Pedaling load was higher in S+F than in C (*d* = 0.274, *p* = 0.004) and S (*d* = 0.254, *p* = 0.005). RPE was lower in S+F than in C (*d* = 0.872, *p* = 0.016) and S (*d* = 0.711, *p* = 0.016).

### 3.3 Sweat loss and absorption

**FIGURE 4:**
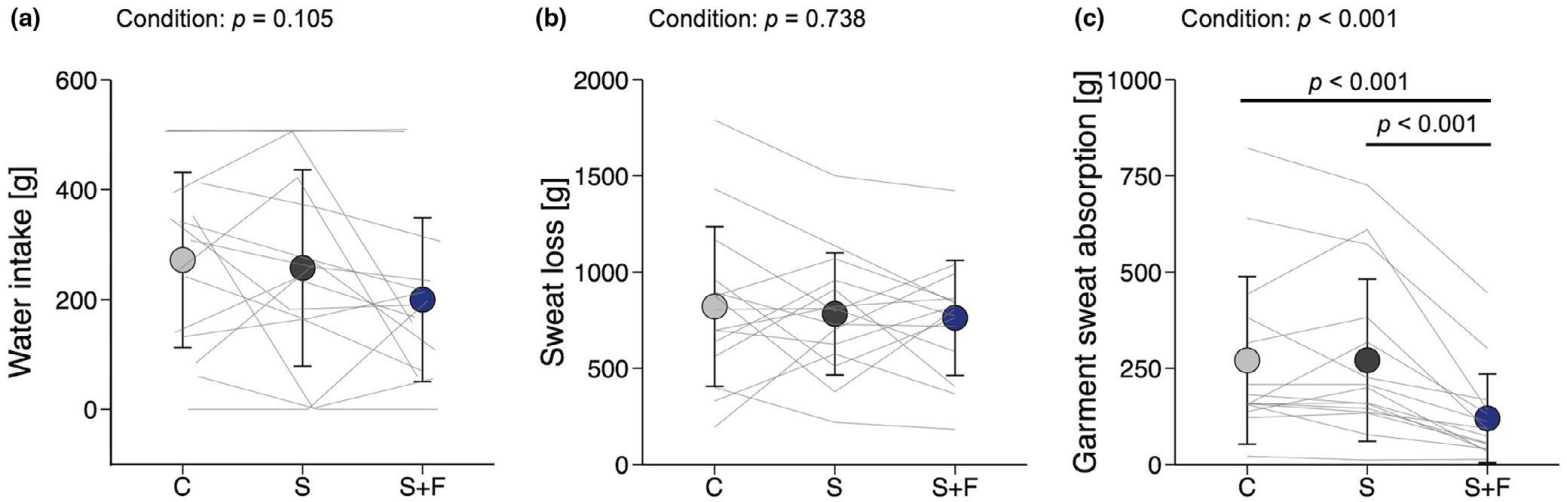
Water intake, total sweat loss, and garment sweat absorption. (a) Water intake and (b) total sweat loss during the experiment. (c) Garment sweat absorption measured at the end of experiment. Data are presented as means and SD with individual line plots (*n* = 15). C, S, and S+F represent wearing base layers made of cross‐shaped fibers, sugar alcohol‐printed base layers, and a combination of S with a fan‐attached jacket, respectively. Data were analyzed with one‐way repeated‐measures analysis of variance followed by paired *t*‐tests with *p* values adjusted by the false discovery rate method.

The ICC and SEM for body mass measured at the beginning of each session across the three experimental days were 1.00 and 0.56 kg, respectively. Figure 4 shows the results for water intake, total sweat loss, and garment sweat absorption (calculated as the difference between wet and dry garment weights). Garment condition had no significant effect on water intake (*η*_*G*_^2^ = 0.038, *p* = 0.105) or total sweat loss (*η*_*G*_^2^ = 0.005, *p* = 0.738). In contrast, garment sweat absorption measured at the end of experiment differed among garment conditions (*η*_*G*_^2^ = 0.134, *p* < 0.001) and was lower in S+F than in C (*d* = 0.867, *p* < 0.001) and S (*d* = 0.890, *p* < 0.001).

### 3.4 Body temperature

**FIGURE 5:**
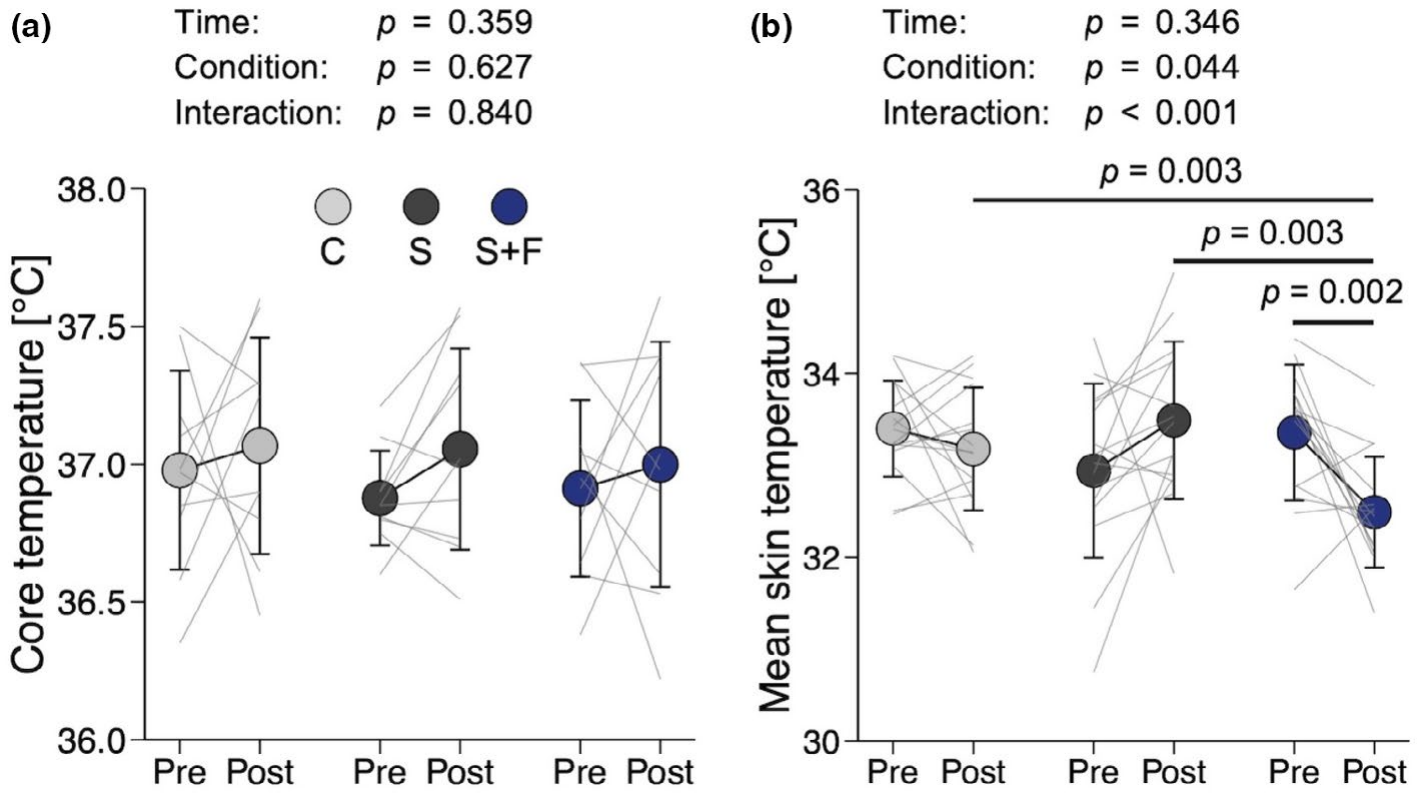
Body temperature. (a) Core temperature, measured on the forehead with a zero‐heat‐flow method. (b) Seven‐site mean skin temperature. Data are presented as means and SD with individual line plots. Core temperature measurements were incomplete in five participants, owing to device malfunction under hot and humid conditions; therefore, *n* = 10 for (a), whereas *n* = 15 for (b). Pre and Post indicate before and after exercise, respectively. C, S, and S+F represent wearing base layers made of cross‐shaped fibers, sugar alcohol‐printed base layers, and a combination of S with a fan‐attached jacket, respectively. Data were analyzed with two‐way repeated‐measures analysis of variance followed by paired *t*‐tests with *p* values adjusted by the false discovery rate method.

Figure 5 shows the results for body temperature. Core temperature, measured on the forehead with a zero‐heat‐flow method, was not significantly affected by time or condition (two‐way ANOVA, time effect, *η*_*G*_^2^ = 0.030, *p* = 0.359; condition effect, *η*_*G*_^2^ = 0.008, *p* = 0.627; time‐by‐condition interaction, *η*_*G*_^2^ = 0.004, *p* = 0.840). The ICC and SEM for the pre‐exercise core temperature measured over 3 days were 0.64 and 0.18°C, respectively. Regarding the seven‐site mean skin temperature, the two‐way ANOVA revealed a main effect of condition (*η*_*G*_^2^ = 0.047, *p* = 0.044) and a time‐by‐condition interaction (*η*_*G*_^2^ = 0.141, *p* < 0.001) but not a main effect of time (*η*_*G*_^2^ = 0.016, *p* = 0.346). Post‐exercise skin temperature in S+F was lower than the pre‐exercise value (*d* = 1.285, *p* = 0.002), as well as the post‐exercise values in C (*d* = 1.083, *p* = 0.003) and S (*d* = 1.348, *p* = 0.003). The ICC and SEM for the pre‐exercise mean skin temperature measured over 3 days were 0.40 and 0.59°C, respectively.

### 3.5 Subjective sensations

**FIGURE 6:**
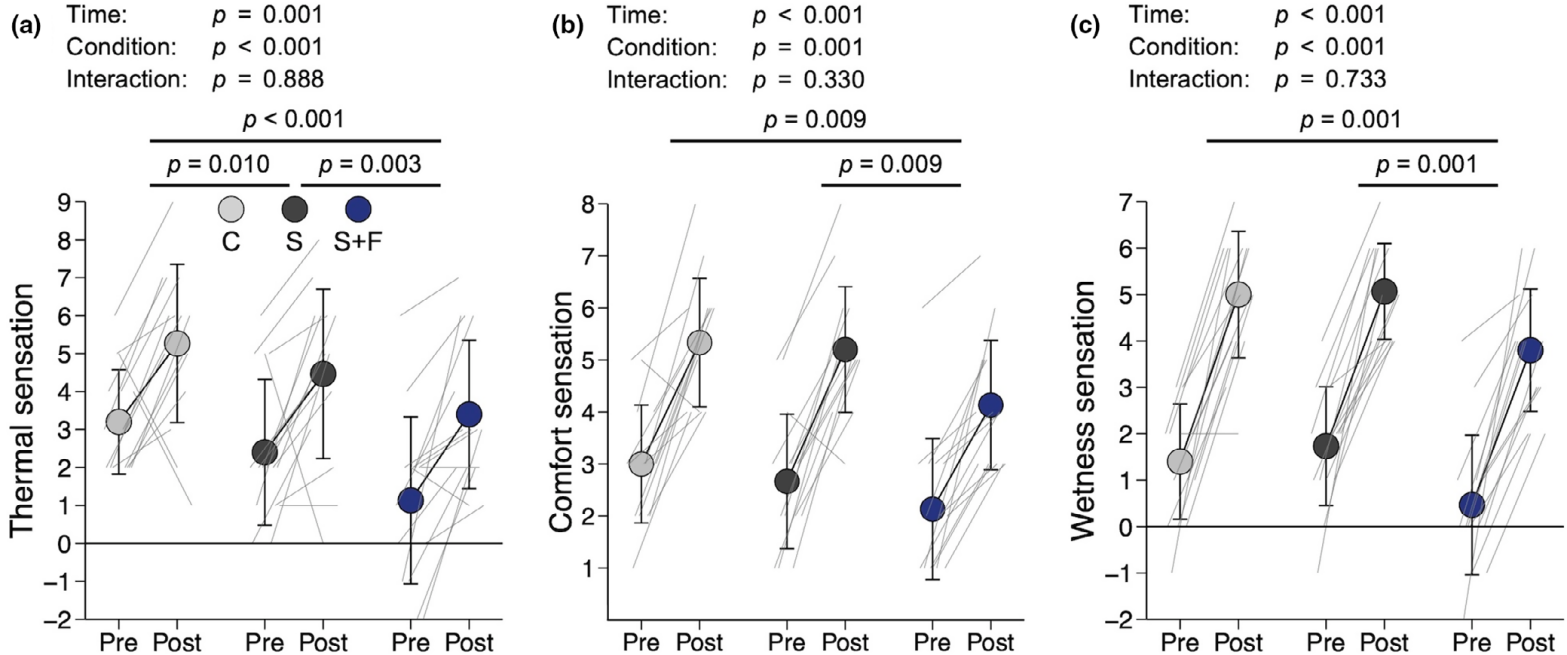
Subjective sensations. (a) Thermal sensation. (b) Comfort sensation. (c) Wetness sensation. Data are presented as means and SD with individual line plots (*n* = 15). Pre and Post indicate before and after exercise, respectively. C, S, and S+F represent wearing base layers made of cross‐shaped fibers, sugar alcohol‐printed base layers, and a combination of S with a fan‐attached jacket, respectively. Data were analyzed with two‐way repeated‐measures analysis of variance followed by paired *t*‐tests with *p* values adjusted by the false discovery rate method.

Figure 6 shows the results for subjective sensations. Ratings of thermal, comfort, and wetness sensations increased after exercise, indicated by significant main effects of time (thermal, *η*_*G*_^2^ = 0.237, *p* = 0.001; comfort, *η*_*G*_^2^ = 0.475, *p* < 0.001; wetness, *η*_*G*_^2^ = 0.651, *p* < 0.001). The two‐way ANOVA also revealed significant main effects of garment condition (thermal, *η*_*G*_^2^ = 0.151, *p* < 0.001; comfort, *η*_*G*_^2^ = 0.119, *p* = 0.001; wetness, *η*_*G*_^2^ = 0.164, *p* < 0.001) but no significant time‐by‐condition interactions (thermal, *η*_*G*_^2^ = 0.001, *p* = 0.888; comfort, *η*_*G*_^2^ = 0.008, *p* = 0.330; wetness, *η*_*G*_^2^ = 0.003, *p* = 0.733). All ratings were lower in S+F than in C (thermal, *d* = 0.896, *p* < 0.001; comfort, *d* = 0.627, *p* = 0.009; wetness, *d* = 0.482, *p* = 0.001) and S (thermal, *d* = 0.502, *p* = 0.003; comfort, *d* = 0.468, *p* = 0.009; wetness, *d* = 0.597, *p* = 0.001). Additionally, thermal sensation was lower in S than in C (*d* = 0.369, *p* = 0.010).

### 3.6 Physical and cognitive performance

Table 1 summarizes the results of the physical and cognitive performance tests, including the vertical jump, chair stand, visual reaction, and the Stroop tests. Within‐day ICC and SEM between the three consecutive measurements (two for the Stroop tests) were 0.97 and 1.2 cm for vertical jump height, 0.89 and 0.44 N kg^−1^ for peak force during rising from the chair, 0.89 and 25 ms for total reaction time, and 0.74 and 68 ms for the Stroop IES, respectively. None of these performance outcomes was affected by garment conditions, as indicated by the absence of a significant main effect of condition or time‐by‐condition interaction. Vertical jump height increased after exercise, whereas RFD in the chair stand test decreased. In the visual reaction test, both total time and premotor times (the vastus lateralis and medial gastrocnemius) decreased after exercise. Between‐day ICC and SEM for pre‐exercise measurements were 0.94 and 1.7 cm for vertical jump height, 0.86 and 0.47 N kg^−1^ for peak force during rising from the chair, 0.85 and 27 ms for total reaction time, and 0.83 and 49 ms for the Stroop IES, respectively.

**TABLE 1:** Physical and cognitive performance outcomes.

		C	S	S+F	ANOVA	*η*_*G*_^2^ value	*p* value
					Factor		
*Vertical jump*							
Height	Pre	35.6 (7.4)	35.9 (6.8)	35.3 (6.7)	**Time**	**0.004**	**0.006**
[cm]	Post	36.4 (6.7)	37.2 (6.9)	35.7 (7.2)	Condition	0.004	0.076
					Interaction	0.001	0.404
*Chair stand*							
Peak force	Pre	17.1 (1.3)	17.3 (1.4)	17.2 (1.1)	Time	<0.001	0.589
[N kg^−1^]	Post	17.1 (1.3)	17.3 (1.3)	17.1 (1.1)	Condition	0.004	0.279
					Interaction	<0.001	0.893
RFD	Pre	127 (13)	134 (21)	130 (18)	**Time**	**0.015**	**0.005**
[N kg^−1^ s^−1^]	Post	123 (15)	126 (19)	129 (17)	Condition	0.014	0.163
					Interaction	0.006	0.254
*Visual reaction*							
Total time	Pre	343 (66)	350 (74)	348 (66)	**Time**	**0.008**	**0.002**
[ms]	Post	327 (61)	338 (78)	340 (71)	Condition	0.004	0.473
					Interaction	0.001	0.620
Premotor time (VL)	Pre	190 (43)	204 (43)	189 (43)	**Time**	**0.006**	**0.028**
[ms]	Post	181 (32)	189 (53)	193 (42)	Condition	0.012	0.220
					Interaction	0.009	0.137
Premotor time (MG)	Pre	170 (38)	177 (40)	171 (37)	**Time**	**0.008**	**0.010**
[ms]	Post	164 (36)	166 (44)	167 (44)	Condition	0.002	0.775
					Interaction	0.002	0.708
*Stroop*							
IES [ms]	Pre	604 (129)	586 (130)	588 (91)	Time	0.003	0.350
	Post	581 (111)	578 (98)	586 (116)	Condition	0.002	0.453
					Interaction	0.002	0.726
Interference IES	Pre	72 (89)	73 (76)	89 (61)	Time	0.003	0.423
[ms]	Post	92 (86)	78 (50)	88 (98)	Condition	0.005	0.606
					Interaction	0.003	0.669

*Note*: Values are means and SD (*n* = 15). Peak force and rate of force development (RFD) were normalized to body mass. Pre and Post indicate before and after exercise, respectively. C, S, and S+F represent wearing base layers made of cross‐shaped fibers, sugar alcohol‐printed base layers, and a combination of S with a fan‐attached jacket, respectively. Significant main effects in two‐way repeated‐measures analysis of variance (ANOVA) are shown in bold (*p* < 0.05).

Abbreviations: IES, inverse efficiency score; MG, medial gastrocnemius; VL, vastus lateralis.

## 4 DISCUSSION

Cooling garments have been developed to mitigate heat strain. However, evidence for their effectiveness on endurance performance in hot and humid environments is limited, with most studies focusing on specific garment types and very light‐to‐moderate physical activity. Moreover, their effects on strength, power, and cognitive performance remain unclear. In this study, we compared the impact of the following three cooling garment conditions on vigorous, heart‐rate‐clamped cycle ergometer performance, as well as on various physical and cognitive performance outcomes in a hot and humid environment: base layers made of cross‐shaped fibers (C), sugar alcohol‐printed base layers (S), and a combination of S with a fan‐attached jacket (S+F). The main finding was that S+F achieved a higher pedaling load while simultaneously producing lower RPE and thermal discomfort than the other conditions. In contrast, the magnitude of post‐exercise fatigue, assessed using various physical and cognitive performance tests, did not differ significantly across conditions.

### 4.1 Cycle ergometer performance and perceived sensation were improved with a combination of sugar alcohol‐printed base layers with a fan‐attached jacket

It is well documented that an increase in body temperature leads to blood flow redistribution toward the skin to promote heat dissipation, which may reduce stroke volume, ultimately impairing endurance performance (Périard et al., 2021; Rowell, 1974). In this study, participants performed a 20‐min cycle ergometer exercise in a well‐controlled hot (~30°C) and humid (~60% relative humidity) environment, with heart rate clamped at 65% of heart rate reserve. As a result, S+F achieved the highest pedaling load with the lowest RPE among the three conditions (Figure 3). These results can be interpreted as improved endurance performance that likely reflects attenuation of skin temperature elevation. Despite a 7% greater pedaling load in S+F than in the other conditions, post‐exercise mean skin temperature was lowest in S+F (Figure 5b), with post‐exercise core temperature remaining comparable across garment conditions (Figure 5a). Previous studies have also reported fan‐attached garments attenuated rectal and esophageal temperature elevations during moderate‐intensity exercise (up to 50% of heart rate reserve) at a constant load (Hadid et al., 2008; Hashimoto et al., 2021; Mori et al., 2022). The decrease in post‐exercise mean skin temperature in S+F (Figure 5b) was likely attributable to efficient evaporative heat loss facilitated by increased airflow from the fans integrated into the jacket. Despite comparable total sweat loss among conditions (Figure 4b), garment sweat absorption at the end of experiment was lowest in S+F (Figure 4c), which indicates the greatest sweat evaporation. This observation is consistent with increased airflow from the fans in S+F. Previous studies have shown that the air velocity around the body is directly associated with evaporative heat loss (Otani et al., 2018, 2021; Saunders et al., 2005).

Subjective ratings of thermal, comfort, and wetness sensations were most favorable in S+F (Figure 6), consistent with previous findings that wearing fan‐attached garments improved thermal and comfort sensations (Hashimoto et al., 2021; Otani et al., 2024; Xu et al., 2025). In addition, S elicited a cooler sensation than C, even without fan assistance (Figure 6a). Nonetheless, this difference in thermal sensation was insufficient to significantly influence cycle ergometer performance. The base layers used in C are designed to provide a cooling effect through efficient sweat absorption and evaporation enabled by their large fiber surface area (Yang et al., 2021). In the present study, however, the participants experienced similarly substantial sweating in both C and S (Figure 4b), which might have contributed to the negligible difference in wetness sensation (Figure 6c). These results suggest that garments with high sweat absorption and evaporation efficiency provide limited cooling benefits under conditions of heavy sweating and high humidity.

### 4.2 Physical and cognitive performance was unaffected by cooling garment conditions

In addition to maintaining and enhancing exercise performance in the heat, alleviating exercise‐induced fatigue is also an important function expected of cooling garments, yet this aspect has often been overlooked in previous research. Therefore, we comprehensively assessed post‐exercise fatigue using both physical and cognitive performance measures. Despite differences in pedaling load among conditions, a comparable decline in RFD was observed in the chair stand test (Table 1). One plausible explanation is that S+F elicited the greatest heat production and neuromuscular fatigue during exercise but facilitated rapid recovery during a 5‐min cool‐down (cycle ergometer exercise at 20 W) and a subsequent data collection period preceding the chair stand test (~5 min). This interpretation is partially supported by a recent study (Naito et al., 2023) showing a substantial recovery (decrease) of rectal temperature with a fan‐attached garment 10 min after the termination of cycle ergometer exercise in a hot and humid environment.

Unexpectedly, no significant decline was observed in other physical or cognitive performance. This may be because the exercise intensity was vigorous but not maximal (Garber et al., 2011). For instance, cycle ergometer exercise at intensities ranging from very light to vigorous in a thermoneutral environment has been reported to enhance executive function, as assessed with the Stroop test (Byun et al., 2014; Kujach et al., 2018; Yanagisawa et al., 2010). In addition, vertical jump height increased after cycle ergometer exercise. This improvement is attributable to reduced tendon hysteresis without altering muscle‐tendon stiffness associated with elevated local limb temperature, which can lead to more efficient elastic energy utilization during countermovement actions (Kubo et al., 2005; Sasajima & Kubo, 2025). The post‐exercise decrease in reaction time also agrees with a previous study reporting shorter reaction time associated with exercise‐induced hyperthermia in a hot environment (Aljaroudi et al., 2020). The shortening of premotor time (the elapsed time from visual stimulus to EMG onset) could be partly attributed to increased nerve conduction velocity resulting from elevated body temperature (Rutkove et al., 1997).

### 4.3 Methodological limitations

The present study has several methodological limitations. First, no negative control condition (e.g., nude or non‐cooling garment) was included. Establishing an appropriate control condition in garment research is inherently challenging because heat dissipation and thermal sensation are influenced by multiple interacting factors, such as fabric type, garment design, and fit (Havenith, 2002). Therefore, we focused on comparing the relative effects of different garment conditions. Instead, the present study reports the between‐day reliability and within‐day repeatability (ICC and SEM) of pre‐exercise physiological measurements, which were generally high, except for body temperature. The relatively low between‐day ICCs of core temperature (0.64) and mean skin temperature (0.40) are consistent with a previous report (Shaw et al., 2024) and attributable to both small individual differences and large physiological between‐day variations in body temperature. We could not assess the between‐day reliability of cycle ergometer performance, hydration status, and perceptual thermal sensations under hot and humid conditions, but the above‐mentioned study also reported them to be moderate‐to‐good (Shaw et al., 2024). Thus, it is highly unlikely that the absence of a negative control condition seriously affects the significance of our findings.

Second, we used the zero‐heat‐flow method to noninvasively measure brain temperature, as in some previous studies (Asai et al., 2023; Fukushima et al., 2023; Onitsuka et al., 2020). However, this method is not necessarily considered a standard approach for assessing core temperature during exercise. In fact, brain temperature measured with the zero‐heat‐flow probe was reported to be higher at rest and increase at a slightly slower rate during exercise in a hot and humid environment than esophageal temperature (Teunissen et al., 2011). In addition, heavy sweating after exercise could have interfered with the accurate measurement of skin surface temperature (Priego Quesada et al., 2015), resulting in an inaccurate estimation of core temperature with this method. Therefore, future studies should employ more standard indicators of core temperature (e.g., rectal, gastrointestinal, or esophageal temperature).

Third, we limited the participants to active young males and restricted the exercise duration (excluding warm‐up and cool‐down) to 20 min to ensure participant safety under hot and humid conditions. Inactive, older, and female individuals have been reported to exhibit lower sweat rates than active young males (Périard et al., 2021), potentially reflecting attenuated thermoregulatory responses. While sweating is an essential physiological response that protects the body from heat stress, excessive sweating may also lead to adverse outcomes such as muscle cramps. In the present study, several participants exhibited a body weight loss exceeding 1% despite being allowed to drink water ad libitum. For these individuals, prolonging the exercise duration might have resulted in a body mass loss approaching or exceeding 2%, a commonly cited threshold for an increased risk of heat illness (Sawka et al., 2007). Further research is warranted to extend the generalizability of the current findings to other populations and longer exercise durations.

Fourth, we used cycle ergometer exercise without external airflow as an exercise model to allow frequent and fine load adjustments and to minimize confounding factors. Thus, different garment effects may be observed during outdoor cycling or running with airflow. Nevertheless, the present study provides fundamental insights into cooling garments and suggests the superiority of S+F during physical activity without external airflow (e.g., indoor or non‐locomotive physical activity) under hot and humid conditions.

## 5 CONCLUSION

The combined use of sugar alcohol‐printed base layers and a fan‐attached jacket resulted in a higher workload with lower perceived exertion during vigorous, heart‐rate‐clamped cycle ergometer exercise under hot and humid conditions. Despite comparable sweat loss, this combination exhibited lower garment sweat absorption than the other garment conditions, suggesting improved endurance performance through efficient evaporative heat loss mainly facilitated by increased airflow from the fans.

## AUTHOR CONTRIBUTIONS

**Kazutaka Ota:** Conceptualization; data curation; formal analysis; funding acquisition; investigation; methodology; project administration; software; visualization. **Hiroaki Tamaru:** Conceptualization; data curation; formal analysis; investigation; methodology; project administration; software. **Kazushige Sasaki:** Conceptualization; funding acquisition; investigation; methodology; project administration; resources; supervision; validation.

## FUNDING INFORMATION

This work was financially supported by Liberta Co., Ltd., Grants‐in‐Aid for Scientific Research (KAKENHI) from Japan Society for the Promotion of Science to K.S. (Grant Numbers JP22K11524 and JP25K14583), and Support for Pioneering Research Initiated by the Next Generation from Japan Science and Technology Agency to K.O. (Grant Number JPMJSP2108).

## CONFLICT OF INTEREST STATEMENT

Cooling garments used in this study were provided by Liberta Co., Ltd. However, the funders had no role in the study design, data collection and analysis, preparation of the manuscript, or decision to publish. The authors received no other specific funding for the present work.

## ETHICS STATEMENT

This study was performed in accordance with the *Declaration of Helsinki* without being registered. Approval was granted by the Ethical Review Committee for Experimental Research involving Human Subjects, The University of Tokyo (approval number: 1020).

## CONSENT

Written informed consent was obtained from the participants.

## Data Availability

Data will be made available upon reasonable request.
